# Vision impairment, hearing impairment and functional Limitations of subjective cognitive decline: a population-based study

**DOI:** 10.1186/s12877-023-03950-x

**Published:** 2023-04-14

**Authors:** Ruirui Guo, Xiaotong Li, Mengzi Sun, Yuxiang Wang, Xuhan Wang, Jing Li, Zechun Xie, Nan Yao, Yixue Yang, Bo Li, Lina Jin

**Affiliations:** Department of Epidemiology and Biostatistics, School of Public Health, Jilin University, 1163 Xinmin Avenue, Changchun, 130021 P. R. China

**Keywords:** Vision impairment, Hearing impairment, Dual impairment, Subjective cognitive decline (SCD), SCD-related functional limitations

## Abstract

**Background:**

The association between sensory impairment including vision impairment (VI), hearing impairment (HI), dual impairment (DI) and the functional limitations of SCD (SCD-related FL) are still unclear in middle-aged and older people.

**Methods:**

162,083 participants from BRFSS in 2019 to 2020 was used in this cross-sectional study. After adjusting the weights, multiple logistic regression was used to study the relationship between sensory impairment and SCD or SCD-related FL. In addition, we performed subgroup analysis on the basis of interaction between sensory impairment and covariates.

**Results:**

Participants who reported sensory impairment were more likely to report SCD or SCD-related FL compared to those without sensory impairment (*p* < 0.001). The association between dual impairment and SCD-related FL was the strongest, the adjusted odds ratios (aORs) and 95% confidence interval (95% CI) were [HI, 2.88 (2.41, 3.43); VI, 3.15(2.61, 3.81); DI, 6.78(5.43, 8.47)] respectively. In addition, subgroup analysis showed that men with sensory impairment were more likely to report SCD-related FL than women, the aORs and 95% CI were [HI, 3.15(2.48, 3.99) vs2.69(2.09, 3.46); VI,3.67(2.79, 4.83) vs. 2.86(2.22, 3.70); DI, 9.07(6.67, 12.35) vs. 5.03(3.72, 6.81)] respectively. The subject of married with dual impairment had a stronger association with SCD-related FL than unmarried subjects the aOR and 95% CI was [9.58(6.69, 13.71) vs. 5.33(4.14, 6.87)].

**Conclusions:**

Sensory impairment was strongly associated with SCD and SCD-related FL. Individuals with dual impairment had the greatest possibility to reported SCD-related FL, and the association was stronger for men or married subjects than other subjects.

**Supplementary Information:**

The online version contains supplementary material available at 10.1186/s12877-023-03950-x.

## Introduction

Subjective Cognitive Decline (SCD) is the self-perceived perception of ongoing cognitive decline, and it typically takes the form of a fall in self-perceived memory loss [1, 2]. As an early marker of mild cognitive impairment and dementia, SCD has attracted more and more attention from scientists in recent years [2–4]. The number of people with SCD was increasing along with the proportion of the elderly population increased. Meanwhile, the functional limitations followed by SCD imposed a huge economic burden on both the family and society. According to the U.S. Centers for Disease Control and Prevention in 2015–2016, more than 10% of people aged 45 and older reported SCD [5].

Vision impairment and hearing impairment are also very common in the context of population aging [6–9]. According to the statistics, more than 5 million suffered vision impairment in the world and 5% of global population was affected by hearing impairment [10–13]. Meanwhile, People with dual sensory impairment accounted for 0.2-2% population of the world [14]. Some studies pointed out vision impairment (VI) and hearing impairment (HI) were major risk factors for cognitive decline [15–17]. Dual sensory impairment (DI) was associated with a significantly increased risk of cognitive decline due to lack of vision or hearing sensory compensation compared with single vision or hearing impairment [18]. However, there are also different results on the association between the sensory impairment and cognitive decline, a cohort study of hearing impairment and cognitive function showed that hearing loss accelerated cognitive decline but the association was not found after adjusting for age [19]. In 2015–2016, more than half of participants with SCD reported functional limitations related to SCD [20]. Previous studies examined the association between sensory impairment and cognitive decline and a study among those showed dual impairment was associated with cognitive decline [21–23]. But few studies explored directly association between sensory impairment and functional limitations of subjective cognitive decline. Although a recent study suggested that people with vision impairment were significantly more likely to report SCD-related FL than those without visual impairment [24]. Older people with visual impairment were more likely to suffer from hearing impairment [25]. There is a lack of evidence of association between hearing impairment or dual impairment and SCD-related functional limitations. Therefore, it is extremely important to study the relationship between sensory impairment (HI, VI, DI) and SCD-related functional limitations.

The purpose of this study was to investigate the association between sensory impairment and SCD or SCD-related functional limitations and to comprehensively assess the impact of the former on latter in a large and representative sample of U.S. adults.

## Method

### Data source and study samples

The source of current study data was the Behavioral Risk Factor Surveillance System (BRFSS) established by the Centers for Disease control and Prevention (CDC)[26]. BRFSS is a nationwide, cross-sectional telephone survey that collects annually the data of U.S. residents aged ≥ 18 concerning health-related risk behaviors, chronic health conditions, and use of preventive services. As far, BRFSS has collected data in all 50 states as well as the District of Columbia and three U.S. territories. To increase the sample size, the research used the data from 2019 to 2020. Among two years, there were totally 48 states chose the module of “cognitive decline” in their surveys. The current study adjusted the survey weights as well as combined the data 2019 and the data of 2020 according to the rule of complex weight of CDC to enhance the representativeness of the sample.

**Fig. 1:**
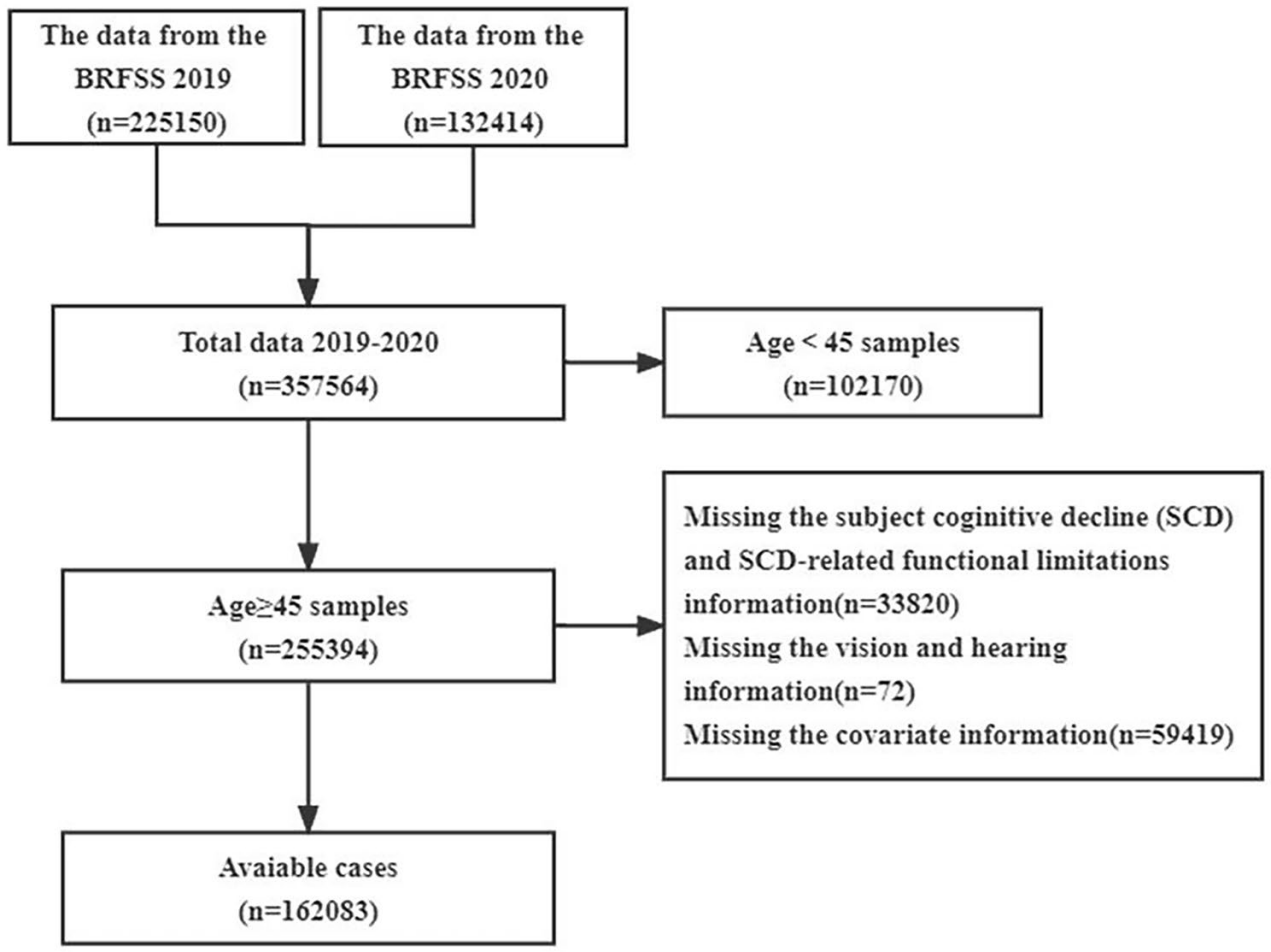
Flow diagram of the sample selection

In our study, the initial database included 357,564 samples during 2019–2020. After excluding individuals aged less than 45, lacking information on SCD and SCD-related limitations, vision and hearing impairment and covariate, 162,083 individuals were included the study (Fig. 1).

### Measures

BRFSS questionnaire included three parts: core questions (for all states in the US), optional modules and state-added questions. “Cognitive decline” and “Dual impairment (vision impairment and hearing impairment)” were included respectively in an optional module and a core question.

Among all respondents aged ≥ 45, those answered “yes” for “During the past 12 have you experienced confusion or memory loss that is happening more often or is getting worse?” was defined SCD. On this basis, those were classified as SCD-related functional limitations if they answer “usually”, “always” or “sometimes” for one or more of follow-up two questions: “During the past 12 months, as a result of confusion or memory loss, how often have you given up day-to-day household activities or chores you used to do (e.g., cooking, cleaning, taking medications, driving, or paying bills)?”and “During the past 12 months, how often has confusion or memory loss interfered with your ability to work, volunteer, or engage in social activities outside the home?”; On the contrary, those answered “rarely”, “never” were defined as no SCD-related functional limitations.

### Sensory impairment (VI, HI, DI)

Respondents who replied “yes” for the question “Are you blind or do you have serious difficulty seeing, even when wearing glasses?” was regarded as vision impairment. Hearing was accessed through the question: “Are you deaf or do you have serious difficulty hearing?”, respondents had a yes answer were defined as hearing impairment. Respondents who had both vision impairment and hearing impairment were defined as dual impairment.

### Covariates

The present study included variables that may confound the association between dual impairment and subjective cognitive decline. Demographic variables included age group (45–64, 65–74, ≥ 75), sex (men or women), race (non-Hispanic white, non-Hispanic Black, non-Hispanic multiracial, Hispanic, non-Hispanic other), marital status (married or other), body mass index(BMI)(underweight [< 18.5], normal weight [18.5–24.9], overweight [25-29.9], obese [≥ 30]), educational level (less than high school graduate, high school graduate/some college, college graduate) and income level (< 15,000, 15,000–25,000, 25,000–35,000, 35,000–50,000, ≥ 50,000). Behavioral and health status variables included smoking status (yes or no), drinking status (yes or no), exercise (yes or no), any chronic diseases (yes or no). Chronic diseases included diabetes, angina or coronary heart disease, stroke and cancer. Among those, BMI was calculated based on self-reported height and weight.

### Statistical analysis

In our analysis, we described the distribution of all samples by three subgroups. All variables were presented with weighted percentages and the corresponding 95% confidence interval (CI). Comparisons between different groups were performed using chi-square test. Multivariate logistic regression with complex weighting was used to estimate the association of sensory impairment with SCD or SCD-related functional limitations and explore interaction between sensory impairment and covariates. We built three models to calculate and report odds ratios (OR) of the outcomes, with the corresponding 95% CI. Model I did not include any covariables. Model II adjusted for age and sex. Model III adjusted for all covariables (age, sex, marital status, BMI, educational level, income level, smoking status, exercise and chronic diseases). The above analyses were performed using IBM SPSS version 24.0. In addition, subgroup analysis plot was conducted using forest plot package for R Version 4.1.1 (R Foundation for Statistical Computing, Vienna, Austria). All *p* value was two-sided, and *p* < 0.05 was considered statistically significant.

## Results

The baseline characteristics of all populations and groups classified by with or without SCD and SCD-related functional limitations are shown in Tables 1 and 2. Among 162,083 individuals, 15,706(9.7%) participants had SCD and 6524(4.0%) had SCD-related FL. In comparison with individuals reported no SCD, a higher proportion of individuals of SCD with or without SCD-related FL reported sensory impairment (HI:7.3%vs19.0%vs15.7%, VI:3.8%vs6.5%vs17.1%, DI:1.0%vs2.6%8.8%). In addition, participants reported SCD-related FL were more likely to be women, smoker, unmarried, no exerciser, people with any chronic disease than the other two groups(*p* < 0.001).

**Table 1:** Basic characteristics of the study population after weighting

Variable level	No.	W % (95%CI)
Age group		
45–64	81,913	61.6(61.1,62.2)
65–74	48,313	23.7(23.2,24.2)
≥ 75	31,857	14.7(14.3,15.1)
**Sex**		
Men	74,631	49.4(48.8,50.0)
Women	87,452	50.6(50.0,51.2)
**Race**		
White, NH	132,829	72.4(71.8,73.1)
Black, NH	11,688	11.4(11.0,11.8)
Multiracial, NH	6203	5.0(4.5,5.5)
Hispanic	2761	1.0(0.9,1.1)
Other, NH	8602	10.2(9.7,10.7)
**Marital status**		
Married	91,301	60.8(60.2,61.3)
Other	70,782	39.2(38.7,39.8)
**BMI**		
Underweight	2198	1.3(1.2,1.5)
Normal Weight	43,747	25.2(24.7,25.8)
Overweight	60,151	37.8(37.2,38.4)
Obese	55,987	35.7(35.1,36.3)
**Educational level**		
Less than high school graduate	9330	11.1(10.7,11.6)
High school graduate/some college	87,256	57.5(56.9,58.1)
College graduate	65,497	31.3(30.8,31.9)
**Income level**		
<15,000	13,773	8.9(8.5,9.2)
15,000–25,000	25,058	15.1(14.7,15.5)
25,000–35,000	16,716	9.7(9.4,10.1)
35,000–50,000	22,987	13.2(12.7,13.6)
≥ 50,000	83,549	53.2(52.6,53.8)
**Smoking status**		
Yes	21,246	14.5(14.1,14.9)
No	140,837	85.5(85.1,85.9)
**Binge drinking**		
Yes	15,829	11.3(10.9,11.7)
No	146,254	88.7(88.3,89.1)
**Exercise**		
Yes	118,201	71.9(71.4,72.5)
No	43,882	28.1(27.5,28.6)
**Any chronic disease**		
Yes	59,860	35.4(34.8,36.0)
No	102,223	64.6(64.0,65.2)
**Independent variable**		
NSI	136,709	85.7(85.3,86.2)
HI	15,759	8.3(8.0,8.6)
VI	6917	4.5(4.3,4.8)
DI	2698	1.4(1.3,1.6)

SCD, subjective cognitive decline, FL, functional limitations, NO., number, W %, Weight%, CI, confidence interval, NH, non-Hispanic, NSI, no sensory impairment, HI, hearing impairment, VI, vision impairment, DI, dual impairment

**Table 2:** Comparison of basic characteristics the study population was classified with or without SCD and SCD-related FL

Variable level	Without SCD (N=146,377)		SCD, without SCD-related FL (N=9182)		SCD, with SCD-related FL (N=6524)		*p* value
	No.	W % (95%CI)	No.	W% (95%CI)	No.	W% (95%CI)	
**Age group**							<0.001
45-64	74,686	62.1(61.5,62.7)	3401	48.3(45.9,50.7)	3826	67.4(64.6,70.0)	
65-74	43,966	23.7(23.2,24.2)	2877	27.3(25.3,29.3)	1470	18.5(16.3,20.8)	
≥75	27,725	14.1(13.8,14.5)	2904	24.5(22.4,26.7)	1228	14.1(12.1,16.4)	
**Sex**							<0.001
Men	67,362	49.7(49.1,50.4)	4565	48.6(46.2,51.0)	2704	43.7(41.0,46.4)	
Women	79,015	50.3(49.6,50.9)	4617	51.4(49.0,53.8)	3820	56.3(53.6,59.0)	
**Race**							<0.001
White, NH	120,09	72.5(71.8,73.2)	7929	79.1(76.8,81.3)	4801	63.3(60.4,66.1)	
Black, NH	10,454	11.5(11.0,11.9)	491	8.2(7.0,9.6)	743	14.4(12.7,16.2)	
Multiracial, NH	5599	5.0(4.5,5.6)	280	3.5(2.6,4.7)	324	5.6(4.4,7.1)	
Hispanic	2442	0.9(0.9,1.0)	152	0.9(0.7,1.3)	167	2.0(1.3,2.9)	
Other, NH	7783	10.1(9.6,10.6)	330	8.2(6.5,10.4)	489	14.7(12.2,17.7)	
**Marital status**							<0.001
Married	84,119	62.1(61.4,62.7)	4834	55.5(53.1,57.9)	2348	40.7(38.0,43.5)	
Other	62,258	37.9(37.3,38.6)	4348	44.5(42.1,46.9)	4176	59.3(56.5,62.0)	
**BMI**							<0.001
Underweight	1917	1.2(1.1,1.4)	130	1.4(0.9,2.0)	151	3.4(2.4,5.0)	
Normal Weight	39,791	25.4(24.8,25.9)	2410	25.3(23.2,27.5)	1546	22.3(19.9,24.7)	
Overweight	54,833	38.2(37.6,38.8)	3363	36.8(34.5,39.2)	1955	29.9(27.5,32.3)	
Obese	49,836	35.2(34.6,35.8)	3279	36.5(34.3,38.8)	2872	44.4(41.7,47.2)	
**Educational level**							<0.001
Less than high school graduate	7784	10.3(9.8,10.8)	540	11.7(10.1,13.6)	1006	27.0(24.3,29.9)	
High school graduate/some college	77,910	57.2(56.6,57.9)	5190	60.8(58.5,63.1)	4156	59.4(56.6,62.1)	
College graduate	60,683	32.4(31.9,33.0)	3452	27.5(25.6,29.5)	1362	13.7(12.2,15.3)	
**Income level**							<0.001
<15,000	11,106	7.7(7.4,8.1)	856	10.3(8.7,12.2)	1811	30.1(27.4,32.8)	
15000-25000	21,360	14.2(13.7,14.6)	1734	19.0(17.1,21.0)	1964	29.2(26.9,31.7)	
25000-35000	14,737	9.4(9.1,9.8)	1159	12.7(11.1,14.5)	820	11.7(10.2,13.3)	
35000-50000	20,756	13.2(12.8,13.7)	1526	14.6(13.3,16.1)	705	10.1(8.7,11.8)	
≥50,000	78,418	55.4(54.8,56.1)	3907	43.4(41.0,45.7)	1224	18.9(16.8,21.2)	
**Smoking status**							<0.001
Yes	18,151	13.6(13.1,14.0)	1237	15.8(14.3,17.5)	1858	31.8(29.4,34.4)	
No	128,22	86.4(86.0,86.9)	7945	84.2(82.5,85.7)	4666	68.2(65.6,70.6)	
**Binge drinkers**							0.826
Yes	14,359	11.3(10.8,11.7)	888	11.8(10.3,13.5)	582	11.2(9.3,13.4)	
No	132,01	88.7(88.3,89.2)	8294	88.2(86.5,89.7)	5942	88.8(86.6,90.7)	
**Exercise**							<0.001
Yes	108,75	73.5(72.9,74.1)	6224	65.2(62.9,67.5)	3223	47.9(45.2,50.7)	
No	37,623	26.5(25.9,27.1)	2958	34.8(32.5,37.1)	3301	52.1(49.3,54.8)	
**Any chronic disease**							<0.001
Yes	51,271	33.2(32.6,33.8)	4529	49.6(47.2,52.0)	4060	62.6(59.9,65.1)	
No	95,106	66.8(66.2,67.4)	4653	50.4(48.0,52.8)	2464	37.4(34.9,40.1)	
**Independent variable**							<0.001
NSI	126,37	87.9(87.5,88.3)	6565	71.9(69.6,74.1)	3771	58.4(55.6,61.0)	
HI	12,875	7.3(7.0,7.6)	1746	19.0(16.9,21.2)	1138	15.7(13.8,17.9)	
VI	5369	3.8(3.6,4.1)	565	6.5(5.4,7.9)	983	17.1(15.1,19.3)	
DI	1760	1.0(0.9,1.1)	306	2.6(2.1,3.2)	632	8.8(7.5,10.4)	

SCD, subjective cognitive decline, FL, functional limitations, NO., number, W %, Weight%, CI, confidence interval, NH, non-Hispanic, NSI, no sensory impairment, HI, hearing impairment, VI, vision impairment, DI, dual impairment

The result of the association between sensory impairment and SCD or SCD-related functional limitations are showed in Table 3. We found that sensory impairment was associated with SCD and SCD-related functional limitations in the unadjusted model [SCD, without SCD-related FL: HI, 3.18(95%CI, 2.75–3.68), VI, 2.10(95%CI, 1.69–2.60), DI, 3.14(95%CI, 2.45–4.03); SCD, with SCD-related FL: HI, 3.25(95%CI, 2.76–3.84), VI, 6.75(95%CI, 5.69-8.00), DI, 13.19(95%CI, 10.67–16.30)]. The association still existed after adjusting age, sex, race, marital status, BMI, educational level, income level, smoking status, binge drinking, exercise, chronic diseases. The association between the dual impairment and the SCD-related FL was the strongest, followed by vision impairment. Hearing impairment had the weakest association with SCD-related FL. [SCD, without SCD-related FL: HI, 2.49(95%CI, 2.14–2.90), VI, 1.73(95%CI, 1.38–2.16), DI, 2.31(95%CI, 1.78–2.98); SCD, with SCD-related FL: HI, 2.88(95%CI, 2.41–3.43)], VI, 3.15(95%CI, 2.61–3.81), DI, 6.78(95%CI, 5.43–8.47)].

**Table 3:** Multiple logistic regression analyses of association between dual impairment and SCD-related functional limitations

		Model I		Model II		Model III	
**Variable**		OR (95%CI)	*p* value	OR (95%CI)	*p* value	OR (95%CI)	*p* value
**SCD, without SCD-related FL**	**NSI**	reference		reference		reference	
	**HI**	3.18(2.75, 3.68)	<0.001	2.79(2.41, 3.23)	<0.001	2.49(2.14,2.90)	<0.001
	**VI**	2.10(1.69, 2.60)	<0.001	2.05(1.65, 2.55)	<0.001	1.73(1.38,2.16)	<0.001
	**DI**	3.14(2.45, 4.03)	<0.001	2.78(2.14, 3.59)	<0.001	2.31(1.78,2.98)	<0.001
**SCD, with SCD-related FL**	**NSI**	reference		reference		reference	
	**HI**	3.25(2.76, 3.84)	<0.001	3.87(3.28, 4.57)	<0.001	2.88(2.41,3.43)	<0.001
	**VI**	6.75(5.69, 8.00)	<0.001	6.73(5.68, 7.98)	<0.001	3.15(2.61,3.81)	<0.001
	**DI**	13.19(10.67, 16.30)	<0.001	15.16(12.17, 18.87)	<0.001	6.78(5.43,8.47)	<0.001

SCD, subjective cognitive decline, FL, functional limitations, NSI, no sensory impairment; HI, hearing impairment; VI, vision impairment; DI, dual impairment, CI: confidence interval, OR, odd ratio

Model I: did not adjust covariates

Model II: adjusted for age and sex

Model III: Model II + adjusted for race, marital status, BMI, educational level, income level, smoking status, binge drinking, exercise, and any chronic disease

**Fig. 2:**
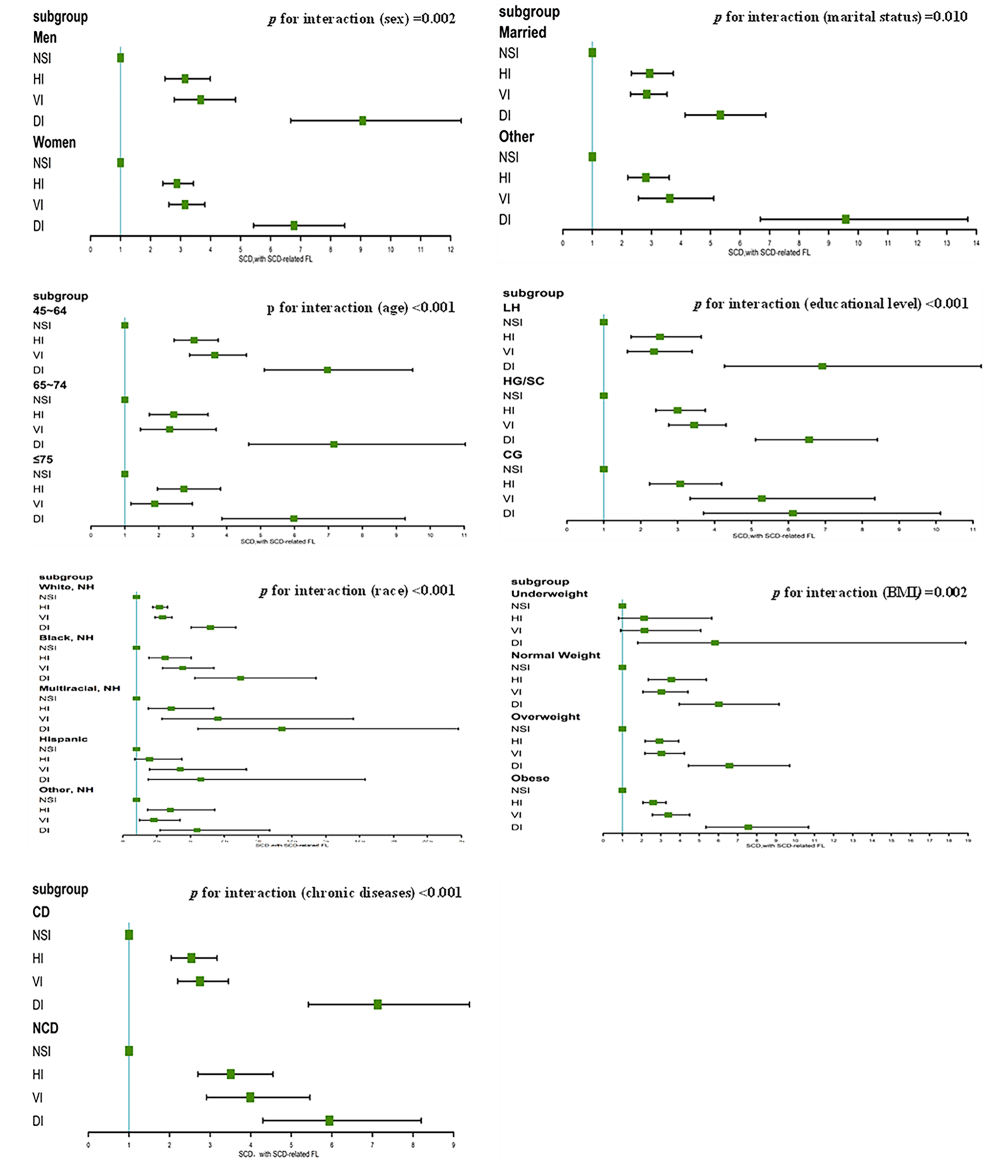
The relationship between dual sensory impairment and SCD, with SCD-related FL according to different subgroups. SCD, subjective cognitive decline, FL, functional limitations, NH, non-hispanic, LH, less than high school graduate, HG/SC, High school graduate/some college, CG, College graduate, NSI, no sensory impairment, HI, hearing impairment, VI, Vision impairment, DI, dual impairment, CD, chronic diseases, NCD, no chronic diseases

The results of the interaction are shown in Fig. 2, and our results indicated that there was an interaction between age or sex or race or BMI or marital status or educational level or chronic diseases and sensory impairment in the association of sensory impairment and SCD-related FL (*p* < 0.05) (Fig. 2). On the basis, we performed subgroups analysis and found that the association between sensory impairment (HI, VI, DI) and SCD-related FL was consistent with all study subjects in subgroups of age, sex, marital status, educational level and chronic diseases (**STable1-4 and 7**). In addition, the result showed that sensory impairment was more significantly associated with SCD-related FL in men than in women [(HI, 3.15(95%CI, 2.48–3.99) vs. 2.69(95%CI, 2.09–3.46); VI, 3.67(95%CI, 2.79–4.83) vs. 2.86(95%CI, 2.22–3.70); DI, 9.07(95%CI, 6.67–12.35) vs. 5.03(95%CI, 3.72–6.81)] **(STable1)**. Among different marital status, the association of dual impairment with SCD-related FL was stronger in married subjects than in other subjects [9.58(95%CI, 6.69–13.71) vs. 5.33(95%CI, 4.14–6.87)] **(STable2)**. In the Underweight group, the association between HI (2.13; 95%CI, 0.80–5.65) or VI (2.15; 95%CI, 0.91–5.08) and SCD-related FL was not obvious (**STable5**).

In addition, we conducted a sensitivity analysis among those without hypertension or depression and discovered that the results were consistent with those of the total population. Sensory impairment was positively correlated with SCD or SCD-related FL. People with dual impairment were more likely to have SCD or SCD-related FL (**STable8, STable9**).

## Discussion

In this study, we found that sensory impairment was strongly associated with SCD and SCD-related functional limitations. Furthermore, the possibility of SCD-related functional limitations was more than twice as high in the presence of dual impairment as in the presence of single sensory impairment. Results of subgroup analysis showed that dual impairment was more strongly associated with SCD-associated FL in men or married subjects than in other subjects.

Our findings were consistent with the results of previous studies which suggested that sensory impairment was relevant to cognitive decline [19, 22, 27–30]. SCD has been thought earliest symptoms of Alzheimer’s Disease (AD) or dementia [31, 32]. Previous studies suggested that sensory impairment was a risk factor for dementia [33], and our study confirmed this view again. Our study indicated the association of sensory impairment and SCD, which was important for the prevention of dementia and Alzheimer’s disease. However, not all people with SCD developed dementia [34], our study demonstrated that sensory impairment should be concerned even in those who were SCD but did not develop dementia. In addition, a former study displayed the association between vision impairment and SCD-related FL [24]. On this basis, our study investigated the association between hearing impairment, dual impairment and SCD-related FL. To our knowledge, this is the first study to examine the relationship between dual impairment and SCD-related functional limitations in middle-aged and older adults. There was a study analyzed the relationship between dual sensory impairment, dementia and functional limitations, then pointed out that dual sensory impairment combined with dementia had a greater impact on daily living functions [35]. In our study, sensory impairment was associated with SCD-related FL and the association was strongest when participants reported dual impairment in our study.

There are several possible explanations for the results: First, long-term loss of sensory input can result in cortical reduction or cortical redistribution like hippocampus, frontal cortex, which may reduce gray matter volume. And gray matter abnormalities have been shown to be closely related to SCD [36–39]. Alternatively, sensory impairment may also affect cognitive function by limiting neural resources associated with cognitive tasks, thereby reducing independence in life [9]. Second, sensory impairment can affect cognitive function through some intermediate variables such as social isolation, low mood, etc.[40]. Third, some common influencing factors (age, vascular disease, the presence of amyloid-β in the lens) led to sensory and cognitive impairment, which together affected life and social function [9, 41–43]. The more common cause was cardiovascular disease and its risk factors, such as high blood pressure, heart disease, smoking, etc., which were closely related to white matter hyperintensity [44–47]. White matter abnormalities have been shown to be associated with cognitive impairment [48–50].

Another important result of our study was that the association between the dual impairment and the SCD-related FL is the strongest, followed by vision impairment. Hearing impairment had the weakest association with SCD-related FL. This may be because the older people with dual impairment had greater functional limitations and lower quality of life than those with single sensory impairment and those with normal sensory function [35, 51]. Vision plays a more important role in everyday tasks than hearing. And vision impairment can be sudden or gradual, whereas hearing impairment tends to be more progressive, so visual impairment was more associated with functional limitation, which has been confirmed by studies [14, 27, 52]. In addition, people with dual impairment were more likely to report SCD-related FL due to lack of compensatory vision or hearing [18].

Although the mechanism regarding the relationship between sensory impairment and SCD or SCD-related FL was not clear, some cohort studies found that some individuals improved cognitive function after cataract surgery after cataract surgery or after the use of hearing-aid [53, 54]. Therefore, recognition and intervention of hearing impairment and visual impairment in the early stage can slow down the rate of cognitive decline and reduce the damage to independent living ability and social ability.

In subgroup analysis, our study also found that men who reported sensory impairment (one or both) were more likely to reported SCD-related FL than women. Our conclusion supported some studies which investigated gender differences in SCD and found that men were far more likely to report SCD than women [55, 56]. In addition, a study on the differences in cognitive function of Intensive Care Unit Survivors indicated that compared with women, although men had a lower risk of cognitive decline, they were more affected by cognitive decline [57]. One possible explanation is that sensory impairment may contribute to social network poverty, which altered brain structure and affected cognitive function by increasing inflammation and glucocorticoid levels. One study showed that social disengagement was more strongly associated with cognitive decline in men than women [43, 58], so men with sensory impairments were more likely to report SCD and related functional limitations. In addition, our study found a stronger link between dual impairment and SCD-related FL in the married people. The reason for this may be that, compared to the unmarried people, married people with disablement were more likely to be cared for by their families, which may aggravate functional limitations [59]. The married with dual impairment may receive less external stimulation including sensory stimulation (taste, skin sensation and so on) due to their family members taking care of them and sharing their household responsibility, which may in turn lead to cognitive decline and SCD-related FL. In addition, more researches may be needed to explore their relationship. This study has some advantages. Firstly, we stratified appropriately and weighted the data and conducted analyses with complex sampling procedures in our work, which can reduce potential bias and increase the reliability of the results. Secondly, our study is the first to explore the association between hearing impairment, dual impairment, and SCD-related functional limitations in middle-aged and elderly people. Thirdly, we conducted a subgroup analysis to observe the relationship between sensory impairment and SCD-related functional limitations in different subgroups.

However, our study has several limitations. Firstly, BRFSS is a sectional study, we could not clear the causal association between sensory impairment and SCD-related functional limitations. Secondly, the BRFSS database collects information in the form of telephone survey. Since the definition of SCD and sensory disorders are subjective, it could be prone to bias. Thirdly, the BRFSS survey did not include adults living in long-term care facilities, prisons, and other facilities; therefore, the findings cannot be generalized to these populations.

## Conclusions

The study found that sensory impairment was associated with SCD and SCD-related functional limitations. The association was strongest for individuals with dual impairment. In addition, men with sensory impairment (VI or HI or DI) were more likely to report SCD-related functional limitations than women. The married subjects with dual impairment had stronger association with SCD-related FL than unmarried subjects. The compensatory role of another sensation is very important when suffering from a single sensory impairment. We should pay more attention to the middle-aged and older people with sensory impairment especially dual impairment.

## Electronic supplementary material

Below is the link to the electronic supplementary material.

Supplementary Material 1

## Data Availability

The datasets generated during the current study are available in the [BRFSS] repository, [https://www.cdc.gov/brfss].

## List of Abbreviations

SCD: Subjective cognitive decline
SCD-related FL: Functional Limitations of Subjective cognitive decline
VI: Vision impairment
HI: Hearing impairment
DI: Dual impairment
BRFSS: Behavioral Risk Factor Surveillance System
CDC: Centers for Disease control and Prevention
CI: Confidence interval
