# Supplementary material for: Vision impairment, hearing impairment and functional Limitations of subjective cognitive decline: a population-based study
Source: BMC Geriatr. 2023 Apr 14;23:230. doi: 10.1186/s12877-023-03950-x (PMC10103414; doi:10.1186/s12877-023-03950-x)
Supplement: Supplementary file 1 — Supplementary Material 1 [file 12877_2023_3950_MOESM1_ESM.pdf]

STable 1. The association between sensory impairment and SCD or SCD-related FL by sex.

STable 2. The association between sensory impairment and SCD or SCD-related FL by marital status.

STable 3. The association between sensory impairment and SCD or SCD-related FL by age.

STable 4. The association between sensory impairment and SCD or SCD-related FL by educational level.

STable 5. The association between sensory impairment and SCD or SCD-related FL by BMI.

STable 6. The association between sensory impairment and SCD or SCD-related FL by race.

STable 7. The association between sensory impairment and SCD or SCD-related FL by chronic diseases.

STable 8. Sensitivity analysis results of association between dual impairment and SCD-related functional limitations in the population without hypertension.

STable 9. Sensitivity analysis results of association between dual impairment and SCD-related functional limitations in the population without depression.

STable 1. The association between sensory impairment and SCD or SCD-related FL by sex

Variable		Men		Women	
		OR (95%CI)	*p* value	OR (95%CI)	*p* value
**SCD, without SCD-related FL**	**NSI**	(reference)		(reference)	
	**HI**	2.38(2.00,2.84)	< 0.001	2.61(2.01,3.39)	< 0.001
	**VI**	1.80(1.28,2.51)	0.001	1.69(1.25,2.28)	0.001
	**DI**	3.24(2.31,4.54)	< 0.001	1.35(0.96,1.89)	0.082
**SCD, with SCD-related FL**	**NSI**	(reference)		(reference)	
	**HI**	3.15(2.48,3.99)	< 0.001	2.69(2.09,3.46)	< 0.001
	**VI**	3.67(2.79,4.83)	< 0.001	2.86(2.22,3.70)	< 0.001
	**DI**	9.07(6.67,12.35)	< 0.001	5.03(3.72,6.81)	< 0.001

SCD, subjective cognitive decline, FL, functional limitations, CI, confidence interval, NH, non-Hispanic, NSI, no sensory impairment, HI, hearing impairment, VI, vision impairment, DI, dual impairment.

STable 2. The association between sensory impairment and SCD or SCD-related FL by marital status

Variable		Married		Other	
		OR (95%CI)	*p* value	OR (95%CI)	*p* value
SCD, without SCD-related FL	NSI	(reference)		(reference)	
	HI	2.42(2.01,2.93)	< 0.001	2.52(1.98,3.22)	< 0.001
	VI	1.94(1.41,2.68)	< 0.001	1.55(1.14,2.11)	0.005
	DI	3.23(2.17,4.81)	< 0.001	1.71(1.25,2.34)	0.001
SCD, with SCD-related FL	NSI	(reference)		(reference)	
	HI	2.81(2.20,3.60)	< 0.001	2.94(2.32,3.74)	< 0.001
	VI	3.62(2.56,5.11)	< 0.001	2.84(2.29,3.53)	< 0.001
	DI	9.58(6.69,13.71)	< 0.001	5.33(4.14,6.87)	< 0.001

SCD, subjective cognitive decline, FL, functional limitations, CI, confidence interval, NH, non-Hispanic, NSI, no sensory impairment, HI, hearing impairment, VI, vision impairment, DI, dual impairment.

STable 3. The association between sensory impairment and SCD or SCD-related FL by age

Variable		45~64		65~74		≥75	
		OR (95%CI)	*p* value	OR (95%CI)	*p* value	OR (95%CI)	*p* value
SCD, without SCD-related FL	NSI	(reference)		(reference)		(reference)	
	HI	2.64(2.03,3.43)	< 0.001	2.52(2.02,3.13)	< 0.001	2.16(1.65,2.81)	< 0.001
	VI	2.18(1.58,3.02)	< 0.001	1.29(0.94,1.77)	0.120	1.07(0.74,1.53)	0.730
	DI	3.29(2.27,4.77)	< 0.001	2.40(1.35,4.26)	0.003	1.42(1.00,1.99)	0.047
SCD, with SCD-related FL	NSI	(reference)		(reference)		(reference)	
	HI	3.04(2.45,3.75)	< 0.001	2.44(1.72,3.45)	< 0.001	2.74(1.96,3.82)	< 0.001
	VI	3.65(2.91,4.58)	< 0.001	2.32(1.46,3.69)	< 0.001	1.88(1.18,2.99)	0.008
	DI	6.97(5.11,9.48)	< 0.001	7.16(4.65,11.03)	< 0.001	5.98(3.86,9.26)	< 0.001

SCD, subjective cognitive decline, FL, functional limitations, CI, confidence interval, NH, non-Hispanic, NSI, no sensory impairment, HI, hearing impairment, VI, vision impairment, DI, dual impairment.

STable 4. The association between sensory impairment and SCD or SCD-related FL by educational level

Variable		Less than high school graduate		High school graduate/ some college		College graduate	
		OR (95%CI)	*p* value	OR (95%CI)	*p* value	OR (95%CI)	*p* value
**SCD, no SCD-related FL**	**NSI**	(reference)		(reference)		(reference)	
	**HI**	2.13(1.41,3.22)	＜0.001	2.53(2.09,3.05)	＜0.001	2.46(1.82,3.33)	＜0.001
	**VI**	1.72(0.94,3.15)	0.080	1.60(1.22,2.09)	0.001	2.25(1.55,3.27)	＜0.001
	**DI**	2.22(1.30,3.81)	0.004	2.37(1.67,3.36)	＜0.001	2.13(1.40,3.22)	＜0.001
**SCD, with SCD-related FL**	**NSI**	(reference)		(reference)		(reference)	
	**HI**	2.44(1.62,3.66)	＜0.001	3.00(2.41,3.75)	＜0.001	3.07(2.24,4.19)	＜0.001
	**VI**	2.13(1.40,3.23)	＜0.001	3.45(2.76,4.31)	＜0.001	5.28(3.34,8.34)	＜0.001
	**DI**	6.70(3.99,11.26)	＜0.001	6.56(5.11,8.41)	＜0.001	6.12(3.70,10.12)	＜0.001

SCD, subjective cognitive decline, FL, functional limitations, CI, confidence interval, NH, non-Hispanic, NSI, no sensory impairment, HI, hearing impairment, VI, vision impairment, DI, dual impairment.

STable 5. The association between sensory impairment and SCD or SCD-related FL by BMI

Variable		Underweight		Normal Weight		Overweight		Obese	
		OR (95%CI)	*p* value	OR (95%CI)	*p* value	OR (95%CI)	*p* value	OR (95%CI)	*p* value
SCD, without SCD-related FL	NSI	(reference)		(reference)		(reference)		(reference)	
	HI	1.68(0.63,4.46)	0.301	2.42(1.79,3.27)	< 0.001	2.94(2.28,3.80)	< 0.001	2.10(1.71,2.58)	< 0.001
	VI	0.81(0.23,2.80)	0.737	1.75(1.02,3.00)	0.041	1.61(1.14,2.28)	< 0.001	1.84(1.36,2.50)	< 0.001
	DI	1.99(0.25,15.67)	0.515	1.69(1.02,2.80)	0.044	2.70(1.64,4.42)	0.007	2.51(1.78,3.55)	< 0.001
SCD, with SCD-related FL	NSI	(reference)		(reference)		(reference)		(reference)	
	HI	2.13(0.80,5.65)	0.130	3.55(2.35,5.37)	< 0.001	2.93(2.18,3.93)	< 0.001	2.60(2.07,3.27)	< 0.001
	VI	2.15(0.91,5.08)	0.082	3.03(2.07,4.41)	< 0.001	3.03(2.17,4.23)	< 0.001	3.39(2.56,4.50)	< 0.001
	DI	5.82(1.80,18.88)	0.003	6.02(3.96,9.16)	< 0.001	6.57(4.44,9.71)	< 0.001	7.56(5.35,10.69)	< 0.001

SCD, subjective cognitive decline, FL, functional limitations, CI, confidence interval, NH, non-Hispanic, NSI, no sensory impairment, HI, hearing impairment, VI, vision impairment, DI, dual impairment.

STable 6. The association between sensory impairment and SCD or SCD-related FL by race

Variable		White, non-Hispanic		Black, non-Hispanic		Multiracial, non-Hispanic		Hispanic		Other, non-Hispanic	
		**OR (95%CI)**	***p* value**	**OR (95%CI)**	***p* value**	**OR (95%CI)**	***p* value**	**OR (95%CI)**	***p* value**	**OR (95%CI)**	***p* value**
	**NSI**	(reference)		(reference)		(reference)		(reference)		(reference)	
**SCD, without**	**HI**	2.54(2.18,2.96)	< 0.001	1.03(0.58,1.82)	0.919	2.49(2.14,2.90)	0.016	1.86(0.83,4.14)	0.129	1.88(0.93,3.80)	0.078
**SCD-related FL**	**VI**	1.61(1.31,1.98)	< 0.001	1.71(0.98,3.00)	0.060	1.73(1.38,2.16)	0.095	1.12(0.45,2.79)	0.803	1.90(0.81,4.46)	0.140
	**DI**	2.60(1.94,3.97)	< 0.001	1.80(0.86,3.78)	0.121	2.31(1.78,2.98)	0.112	0.42(0.10,1.84)	0.251	1.17(0.49,2.81)	0.726
	**NSI**	(reference)		(reference)		(reference)		(reference)		(reference)	
**SCD, with**	**HI**	2.71(2.22,3.30)	< 0.001	3.13(1.94,5.05)	< 0.001	3.56(1.89,6.71)	< 0.001	1.97(0.89,4.36)	0.096	3.51(1.82,6.77)	< 0.001
**SCD-related FL**	**VI**	2.93(2.36,3.63)	< 0.001	4.42(2.92,6.72)	< 0.001	7.03(2.89,17.01)	< 0.001	4.24(1.98,9.11)	< 0.001	2.28(1.23,4.23)	0.009
	**DI**	6.48(5.04,8.33)	< 0.001	8.70(5.31,14.25)	< 0.001	11.75(5.57,24.78)	< 0.001	5.76(1.86,17.88)	0.002	5.48(2.77,10.85)	< 0.001

SCD, subjective cognitive decline, FL, functional limitations, CI, confidence interval, NH, non-Hispanic, NSI, no sensory impairment, HI, hearing impairment, VI, vision impairment, DI, dual impairment.

STable 7. The association between sensory impairment and SCD or SCD-related FL by chronic diseases

Variable		Any chronic diseases		NO chronic diseases	
		OR (95%CI)	*p* value	OR (95%CI)	*p* value
SCD, without SCD-related FL	NSI	(reference)		(reference)	
	HI	2.30(1.87,2.85)	$<$ 0.001	2.71(2.20,3.34)	$<$ 0.001
	VI	1.17(0.91,1.50)	0.233	2.74(1.95,3.86)	$<$ 0.001
	DI	2.21(1.57,3.11)	$<$ 0.001	2.46(1.65,3.67)	$<$ 0.001
SCD, with SCD-related FL	NSI	(reference)		(reference)	
	HI	2.54(2.04,3.17)	$<$ 0.001	3.51(2.70,4.55)	$<$ 0.001
	VI	2.75(2.20,3.45)	$<$ 0.001	3.99(2.91,5.46)	$<$ 0.001
	DI	7.13(5.42,9.39)	$<$ 0.001	5.94(4.30,8.20)	$<$ 0.001

SCD, subjective cognitive decline, FL, functional limitations, CI, confidence interval, NH, non-Hispanic, NSI, no sensory impairment, HI, hearing impairment, VI, vision impairment, DI, dual impairment.

STable8. Sensitivity analysis results of association between dual impairment and SCD-related functional limitations in the population without hypertension

Variable		Model I		Model II		Model III	
		OR (95%CI)	*P* value	OR (95%CI)	*p* value	OR (95%CI)	*p* value
**SCD, without**	**NSI**	(reference)		(reference)		(reference)	
**SCD-related FL**	**HI**	3.39(2.66, 6.55)	< 0.001	3.01(2.30, 3.93)	< 0.001	2.75(2.12,3.55)	< 0.001
	**VI**	2.35(1.66, 3.32)	< 0.001	2.25(1.59, 3.19)	< 0.001	1.97(1.40,2.79)	< 0.001
	**DI**	4.17(2.66, 6.55)	< 0.001	3.89(2.47, 6.13)	< 0.001	3.04(1.90,4.87)	< 0.001
	**NSI**	(reference)		(reference)		(reference)	
**SCD, with**	**HI**	3.64(2.77, 4.79)	< 0.001	4.31(3.21, 5.79)	< 0.001	3.20(2.40,4.28)	< 0.001
**SCD-related FL**	**VI**	7.11(5.19, 9.74)	< 0.001	7.22(5.28, 9.89)	< 0.001	3.32(2.28,4.84)	< 0.001
	**DI**	14.28(9.34, 21.83)	< 0.001	15.81(10.22, 24.46)	< 0.001	5.96(3.20,3.32)	< 0.001

SCD, subjective cognitive decline, FL, functional limitations, NSI, no sensory impairment; HI, hearing impairment; VI, vision impairment; DI, dual impairment, CI: confidence interval, OR, odd ratio

Model I: did not adjust covariates.

Model II: adjusted for age and sex.

Model III: Model II+ adjusted for race, marital status, BMI, educational level, income level, smoking status, binge drinking, exercise, and any chronic disease.

Stable9. Sensitivity analysis results of association between dual impairment and SCD-related functional limitations in the population without depression

		Model I		Model II		Model III	
**Variable**		**OR (95%CI)**	***P* value**	**OR (95%CI)**	***p* value**	**OR (95%CI)**	***p* value**
**SCD, without**	**NSI**	(reference)		(reference)		(reference)	
**SCD-related FL**	**HI**	3.20(2.62, 3.91)	< 0.001	2.57(2.10, 3.14)	< 0.001	2.32(1.89,2.84)	< 0.001
	**VI**	2.00(1.46, 2.75)	< 0.001	1.92(1.40, 2.65)	< 0.001	1.67(1.21,2.30)	0.002
	**DI**	2.87(2.12, 3.88)	< 0.001	2.29(1.65, 3.18)	< 0.001	2.02(1.49,2.74)	< 0.001
	**NSI**	(reference)		(reference)		(reference)	
**SCD, with**	**HI**	4.25(2.95, 6.14)	< 0.001	4.21(3.06, 5.78)	< 0.001	3.34(2.42,4.62)	< 0.001
**SCD-related FL**	**VI**	7.19(5.35, 9.66)	< 0.001	7.15(5.31, 9.62)	< 0.001	3.62(2.65,4.94)	< 0.001
	**DI**	17.66(11.61, 26.84)	< 0.001	17.48(11.24, 27.18)	< 0.001	8.64(5.95,12.53)	< 0.001

SCD, subjective cognitive decline, FL, functional limitations, NSI, no sensory impairment; HI, hearing impairment; VI, vision impairment; DI, dual impairment, CI: confidence interval, OR, odd ratio

Model I: did not adjust covariates.

Model II: adjusted for age and sex.

Model III: Model II+ adjusted for race, marital status, BMI, educational level, income level, smoking status, binge drinking, exercise, and any chronic disease.
